# One‐Year Analysis of Clinical and Radiological Outcomes of Two‐Piece Zirconia Compared to Titanium Implants: A Multicenter Prospective Randomized Clinical Trial

**DOI:** 10.1111/clr.70094

**Published:** 2026-01-22

**Authors:** Marc Balmer, Michael Payer, Anke Steinwender, Valentin Herber, Ronald E. Jung, Sebastian Kühl

**Affiliations:** ^1^ Clinic of Reconstructive Dentistry, Center for Dental Medicine Zurich University of Zurich Zurich Switzerland; ^2^ Department of Oral Surgery and Orthodontics, University Clinic for Dental Medicine & Oral Health Medical University of Graz Graz Austria; ^3^ Department of Restorative Dentistry, Periodontology and Prosthodontics, University Clinic for Dental Medicine & Oral Health Medical University of Graz Graz Austria; ^4^ Department of Oral Surgery, University Center for Dental Medicine in Basel University of Basel Basel Switzerland

**Keywords:** bone loss, ceramic, dental implants, implant survival, titanium implants, two‐piece implants, zirconia implants

## Abstract

**Objectives:**

To evaluate the clinical performance of two‐piece zirconia implants with screw‐retained abutments compared to titanium implants after 1 year of loading.

**Materials and Methods:**

In this multicenter, prospective, randomized clinical trial, 61 two‐piece zirconia and 61 titanium implants were placed and restored with single crowns. Evaluations were performed at implant placement, crown delivery, and 1‐year post‐loading. Peri‐implant Marginal Bone Loss (MBL), survival rate, early wound healing index, and soft tissue parameters were assessed. Intergroup comparisons of continuous outcomes were performed using Linear Mixed‐Effects Models accounting for center and potential confounders. Categorical variables were analyzed using the chi‐square or Fisher's exact test. Survival was analyzed using Kaplan–Meier estimates. Significance was set at *p* < 0.05.

**Results:**

No significant differences were found between the two implant types. Mean MBL from implantation to crown insertion was 1.10 ± 0.78 mm for titanium and 0.94 ± 0.67 mm for zirconia implants. No significant additional bone loss occurred over the subsequent year, with changes of 0.07 ± 0.55 mm and 0.08 ± 0.51 mm for titanium and zirconia, respectively. After 1 year, zirconia implants showed a 100% survival rate, while titanium implants showed 96.5% with two failures. At 1 year, differences in probing depths, plaque accumulation, and Papilla Bleeding Index were not statistically significant.

**Conclusion:**

After 1 year of loading, no statistically significant differences in MBL, implant survival, or peri‐implant health were found between zirconia and titanium implants, indicating no clinical superiority. Zirconia implants may therefore be considered a viable alternative in single‐tooth implant restorations.

**Trial Registration:**

The study is registered at the German Clinical Trial Register (No. DRKS 00013209) as well as at the Federal Office of Public Health's (FOPH) portal for human research in Switzerland (kofam.ch)

## 1 Introduction

Over the last four decades, dental implants have become the major treatment option for replacing missing teeth. Considering their biocompatibility and mechanical properties like durability, commercially pure titanium (Ti) and its alloy, Ti_6_Al_4_V, have been the most commonly used material for the manufacture of dental implants (Nicholson 2020). However, the color of titanium is grey, giving rise to esthetic problems. It is particularly disadvantageous if the soft tissue situation is not optimal and the dark color shines through the thin peri‐implant mucosa (Kohal et al. 2004). Additionally, studies have reported that metal hypersensitivity reactions like vague pain, skin rashes, fatigue, and malaise might occur upon degradation of metallic biomaterials, including titanium (Siddiqi et al. 2011). Some case reports have suggested that titanium hypersensitivity might result in implant failure (Alqahtani et al. 2021; du Preez et al. 2007). Similarly, another study found that a minority of the population may also show immunologic reactions (Sicilia et al. 2008) that might be an important trigger in the general development and severity of peri‐implant infections (Fretwurst et al. 2016). The patients thus wish for a metal‐free restoration (Andreiotelli et al. 2009). These limitations of titanium implants emphasize the need to use other materials to manufacture dental implants. To this end, alumina‐based dental implants were used initially but exhibit weak biomechanical properties, for example, high risk for fracture (d'Hoedt and Schulte 1989). In contrast to other ceramics, zirconia (ZrO_2_) has improved biomechanical resistance with less fracture tendency and superior bending stress properties (Andreiotelli et al. 2009). Several preclinical and clinical studies have shown similar osseointegration for ZrO_2_ implants compared to titanium (Akagawa et al. 1993; Gahlert et al. 2007, 2010; Sennerby et al. 2005). Some studies have even shown improved biocompatibility for soft tissue and a favorable response to the alveolar bone with zirconia over titanium implants (Bienz et al. 2021; Blaschke and Volz 2006; de Meiros et al. 2013; Haugen and Chen 2022). Therefore, zirconia seems to have a high potential as an alternative material to titanium in dental implants.

One limitation of zirconia‐based dental implants is the connection between the implant and crown. While in titanium, most abutments are screw‐retained, creating a screw‐retained connection is a major challenge in zirconia implants due to material properties. Although safe and reliable to use, most zirconia implants follow a one‐piece design, which limits prosthetic flexibility and makes their use challenging in implant positions requiring angulated abutments (Balmer et al. 2020; Blaschke and Volz 2006; Payer et al. 2013; Pieralli et al. 2017). This limitation can be resolved with the use of two‐piece zirconia implants. Some of the recent data with two‐piece zirconia implants showed no significant difference in the success rates compared to two‐piece titanium implants (Becker et al. 2017; Koller et al. 2020). However, these studies were limited by a low number of subjects. Importantly, these studies involved the use of adhesive or cement‐retained ceramic crowns, which have been associated with an increased incidence of biological complications and may affect implant outcomes (Kraus et al. 2024; Wittneben et al. 2014). Therefore, generating two‐piece ZrO_2_ implants with screw‐retained abutment connections would greatly advance dental implantology.

This study investigates a commercially available two‐piece zirconia implant (Institut Straumann AG, Basel, Switzerland) featuring a screw‐retained abutment. Its design is closely related to the well‐documented two‐piece titanium Tissue Level (TL) implant system, which has demonstrated 10‐year survival rates of up to 95% (Jung et al. 2012). Key differences include a modified shoulder with a straight step to reduce fracture risk and a screw‐retained connection that involves inserting a titanium screw into the zirconia implant body. The implant's tapping corresponds to that of the Bone Level (BL) titanium implant (Institut Straumann AG, Basel, Switzerland), known for higher primary stability. The surface (ZLA: Zirconia Large‐grit Acid‐etched) features macro‐ and micro‐roughness and has shown promising results in preclinical and clinical studies of one‐piece zirconia implants (Straumann Pure Ceramic Implant Monotype; Institut Straumann AG, Switzerland) (Gahlert et al. 2007, 2009, 2010, 2016). While randomized clinical trials with 5‐year data exist comparing these one‐piece zirconia and titanium implants restored with cemented crowns (Ruiz Henao et al. 2024), no randomized studies have yet reported the safety and performance of the two‐piece ZrO_2_ implant with screw‐retained abutment connection.

Therefore, the aim of the study was to assess the clinical outcomes of two‐piece zirconia implants with screw‐retained abutments in comparison to titanium implants after 1 year of functional loading.

## 2 Material and Methods

### 2.1 Study Details

#### 2.1.1 Study Design

The study was conducted as a multicenter, prospective, randomized clinical trial. Three centers involved in the study were (i) Department of Oral Surgery, University Center for Dental Medicine in Basel, University of Basel, Basel, Switzerland (ii) Clinic of Reconstructive Dentistry, Center for Dental Medicine, University of Zurich, Zurich, Switzerland and (iii) Department of Oral Surgery and Orthodontics, University Clinic for Dental Medicine & Oral Health, Medical University of Graz, Graz, Austria.

The study was conducted after approval from the Competent Ethics Committee (CEC). This included a vote from the Ethics Committee of Northwestern and Central Switzerland and the Ethics Committee of the Medical University of Graz in agreement with local requirements. Furthermore, the study was performed in accordance with the German Clinical Trial Register (DRKS 00013209), World Medical Association Declaration of Helsinki, and the International Organization for Standardization guideline ISO 14155 (Clinical investigation of medical devices for human subjects—Good clinical practice). All patients provided informed consent before the start of the study. At all stages of the study, the individual subject's medical information was handled with confidentiality by using subject identification code numbers to correspond to treatment data.

#### 2.1.2 Study Population

The study population comprised patients of or above 18 years of age who provided informed consent and accepted the clinical and radiographic analysis and maintenance required according to the protocol. The study population had single tooth gaps (with at least one adjacent tooth) with a sufficient amount of horizontal and vertical bone, including the need for contour augmentation. Patients with signs of occlusal parafunctions (e.g., bruxers), known or suspected non‐compliance or drug or alcohol abuse, previous irradiation in the neck/head area, and general contraindications against implant treatment or medication potentially compromising osseointegration (e.g., immunodeficiency, advanced systemic diseases, corticosteroid or bisphosphonate medication) were excluded, as well as those with acute periodontal disease. Patients with a history of periodontitis were only included if they had received individualized periodontal therapy tailored to their clinical condition, followed by successful reevaluation prior to study enrollment. Furthermore, heavy smokers (> 10 cigarettes per day), pregnant or breastfeeding women, subjects who did not provide consent or comply with the study protocol, subjects who were unable to follow the procedures of the study, for example, due to language problems, psychological disorders, dementia, etc. of the participant, who were participating in another study with an investigational product during the present study were also excluded. Additionally, as per the secondary exclusion criterion, patients with a lack of primary stability of the implant (hand testing directly after surgery) at or immediately after implant surgery were also excluded. Importantly, patients were withdrawn from the study if an event defined as exclusion criteria occurred during the study or if patients wished to quit the study for any reason.

#### 2.1.3 Study Implants

The control implants used in this study were the titanium Straumann Tissue Level Implant Standard Plus with a diameter of 4.1 mm, a minimum length of 8 mm, and a prosthetic platform of 4.8 mm (Regular Neck [RN]) (Institut Straumann AG, Basel, Switzerland). The surface of these implants was sandblasted and acid‐etched (SLA), giving the implant's surface a microstructure of 1–5 μm and a macrostructure of 40 μm. These implants had a transmucosal part of 1.8 mm with a machined surface (Standard Plus, SP). Abutments can be screw‐retained in a platform with a skewed margin.

The test implants used in this study were Straumann PURE Ceramic Implant with similar configurations as the control implants, specifically, a diameter of 4.1 mm, a minimum length of 8 mm, and a prosthetic platform of 4.8 mm (RN) (Institut Straumann AG, Basel, Switzerland). These implants were made of zirconia and had a similar surface structure that was also sandblasted and acid‐etched (ZLA) with almost identical micro‐ and macrostructure. As the control implants, test implants also had a 1.8 mm transmucosal machined part (Standard Plus, SP). Abutments may be screw‐retained with a flat platform.

Both implants are CE‐certified and were placed only by dentists trained for the study. No specific additional knowledge is required for the test implant compared to the control implant.

#### 2.1.4 Study Protocol and Surgical Procedure

**FIGURE 1:**
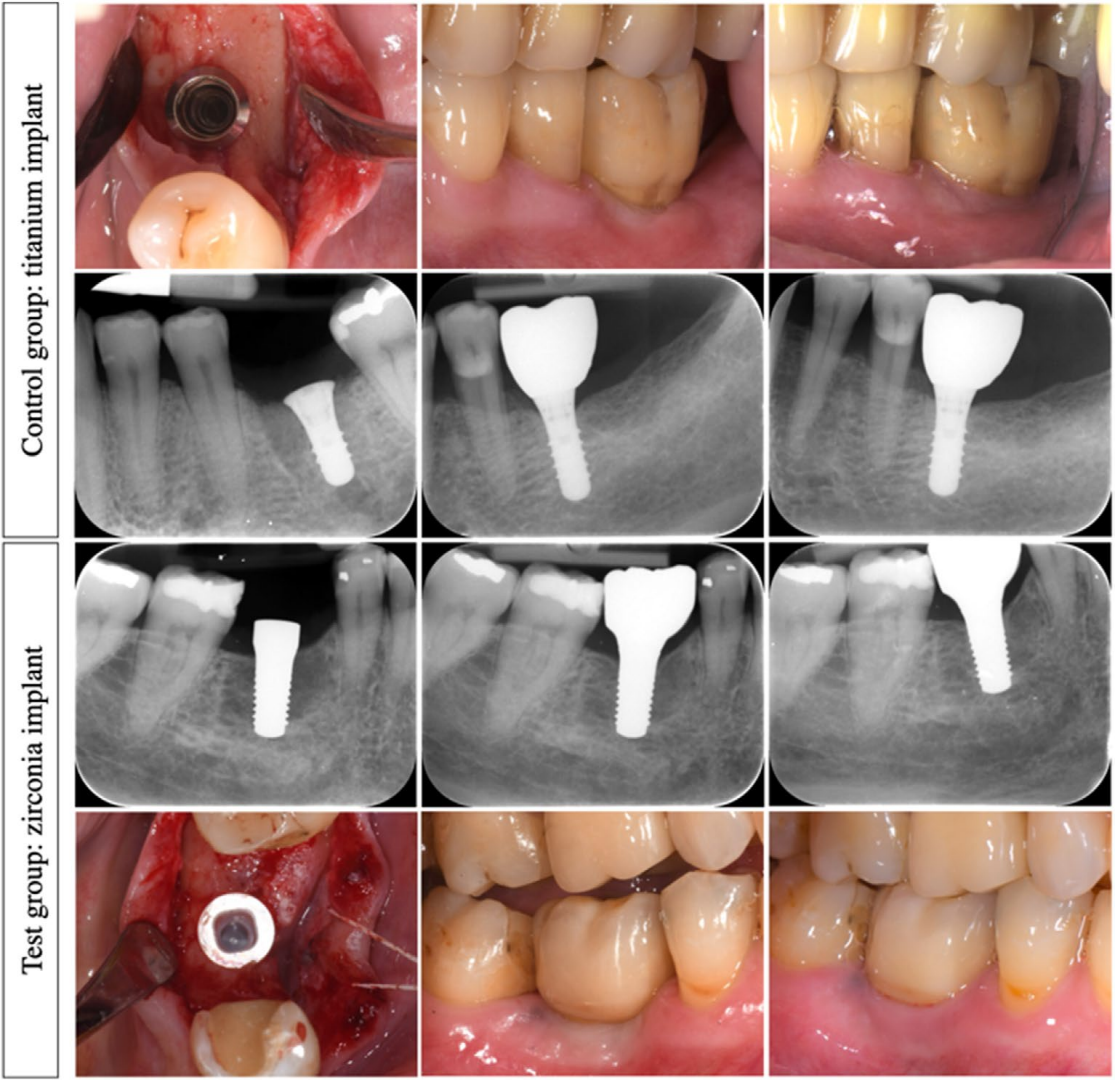
A series of clinical photos and radiographic images from both the test and control groups obtained at the time of implant placement, crown insertion, and at the 1‐year follow‐up.

**FIGURE 2:**
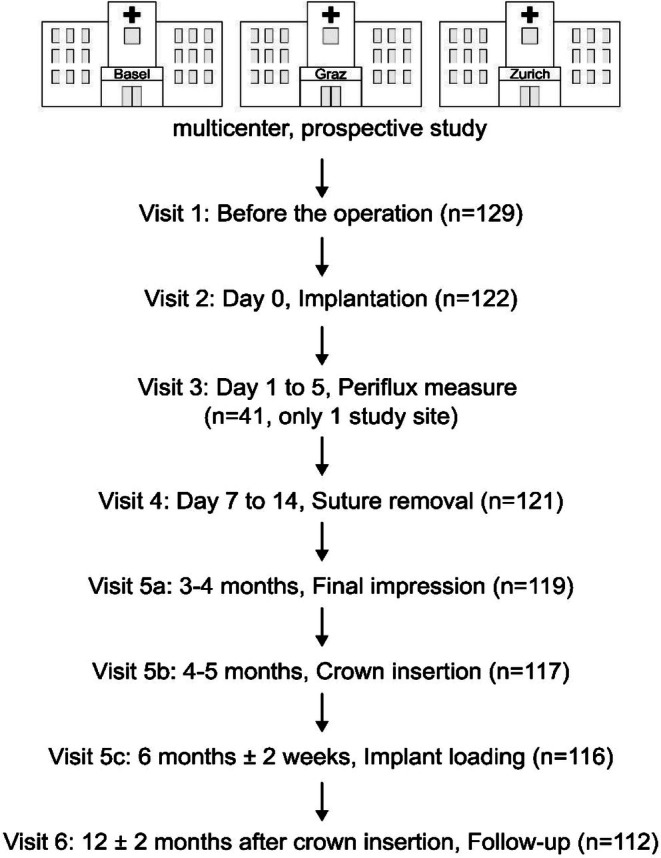
An outline of the study centers, timeline of patient visits, and patient number at each visit as pooled data from all three centers.

In cases with limited bone volume, Cone Beam Computed Tomography (CBCT) was performed to estimate the bone height and for guided implant placement. Implants were placed at least 10 weeks after tooth extraction, representing the early phase of a Type 3 placement, characterized by implant insertion following partial healing of soft and hard tissues (Hämmerle et al. 2004). For implantation, a mid‐crestal incision was performed on top of the alveolus after local anesthesia according to the general procedure. This was chosen in a way that a sufficient band of keratinized tissue was ensured on the buccal aspect of the placed implant. The incision was followed by a marginal incision around the neighboring teeth. The bone was exposed, raising a full‐thickness flap. After raising a full mucoperiosteal flap, drills of increasing diameter were applied (starting with rose burs and continuing with the twisted drill with 2.2, 2.8, and 3.5 mm diameter and a depth of 8 mm or 10 mm depending on the available vertical amount of bone). Before inserting the implant, the respective profile drill was additionally applied. A distance of 2 mm was respected to anatomical critical structures (nerve and blood vessels) according to standard recommendations. In the cases of hard bone (class one), a tap (tapper of the bone level system) was drilled before implant insertion. The insertion depth was determined individually based on the planned prosthetic position of the final crown. The restorative margins were epimucosally in the non‐esthetic zone and submucosally in the esthetic zone. Following these prosthetic considerations, subcrestal positioning of the rough–smooth transition line was permitted. Whether a situation may be regarded as an esthetic zone or not was evaluated individually according to the patient's anatomy. Implants were inserted with torque control to ensure a torque of > 10 Ncm (primary stability). In cases of contour augmentation, local bone chips were collected, applied on the micro‐rough implant structure, and covered by a bone substitute material and a collagen membrane (BioGide, Geistlich, Pharma AG, Wolhusen, Switzerland), generally known as Guided Bone Regeneration (GBR). In all cases, transmucosal and submerged healing were performed. The healing cap was placed 1 mm above the gingival margin to avoid pseudo‐pockets. After implant insertion and suture closure, control radiography was performed (single radiography in parallel technique). One week after implant insertion, sutures were removed. Three months after implant insertion, conventional or digital impressions were taken. Implants were loaded 4 months after implant insertion with screw‐retained crowns (made of lithium disilicate or zirconia, veneering was allowed) and PURE or standard titanium bases. Crowns were fixed with 35 Ncm torque. After crown insertion, radiographies (single radiography parallel technique) were performed to control the crown fit and estimate the bony situation. Single radiographies were repeated 1 year after implant insertion with the same technique (Figure 1). All radiographies correspond to the general approach and no image was performed only for study reasons. Figure 2 summarizes the timeline of the study protocol. A detailed description of the patient number is provided in the results section.

### 2.2 Data Measurement and Analysis

#### 2.2.1 Assessment of Primary Outcome

The primary outcome variable of the study was the marginal vertical bone change in millimeters (mm) 1 year after crown insertion. A single radiography was used in a parallel technique. Measurements were performed using ImageJ (ImageJ 1.50i, National Institutes of Health, Bethesda, MD, USA). Radiographic images were calibrated based on the known distance between the implant thread height. Then the distance between the implant shoulder and the crestal bone was determined mesial and distal of the implant. The mean of both values was calculated and used to determine the marginal bone level at each time point. In this study, both the control and test implants had the same polished shoulder height (1.8 mm) and, consequently, the same distance from the shoulder to the transition between the smooth and rough surfaces. This data was subtracted from the identical measurement and calculation of the initial radiography after implant insertion. This way, the mesial and distal bone remodeling was monitored after 1 year, and the effective bone loss over time was calculated. All marginal bone level measurements were performed by a single examiner. Blinding was not feasible due to the clearly distinguishable radiographic characteristics of the two implant types.

#### 2.2.2 Assessment of Secondary Outcomes

The secondary objectives of the study focused on comparing the early wound healing index (EWHI) (Wachtel et al. 2003) between control and test implant recipients at suture removal (day 7–12, visit 4). The plaque index (PI) (Silness and Löe 1964) and mesial and distal papilla bleeding indexes (PBI) (Saxer and Mühlemann 1975) were measured at implant loading and 1 year after crown insertion. Probing depth was measured 1 year after crown insertion. Overall implant survival was also calculated after 1 year of implantation. In each center, all clinical assessments were performed by a single examiner. Prior to study initiation, an inter‐examiner calibration session was conducted to ensure consistency across centers. Blinding was not feasible due to the clearly distinguishable clinical appearance of the two implant materials.

#### 2.2.3 Assessment of Safety Outcomes

Adverse events were recorded at each visit and intervention. Any adverse event was directly communicated personally (by telephone) and written between the responsible investigators of the three participating centers as well as the implant manufacturer. In case of unexpected events such as fractures of material compounds, the reason for the fracture was planned to be investigated in labs by Energy‐Dispersive X‐ray Spectroscopy (EDX) and Scanning Electron Microscopy (SEM) analyses at the University of Basel.

#### 2.2.4 Statistics

The study was designed as a superiority trial to detect a clinically relevant difference in marginal bone remodeling between a two‐piece zirconia implant with a screwed abutment and a conventional titanium implant. The null hypothesis stated that there would be no difference in marginal bone remodeling between the two implant systems.

The required sample size was determined through an initial power calculation to achieve at least 80% power to detect a statistically significant difference between groups. For calculation, the mean value for bone remodeling and standard deviation for the control implant (Calvo‐Guirado et al. 2016) was compared to the one‐piece zirconia implant (Gahlert et al. 2016), providing identical intraosseous surface designs for the respective test implant. A sample size of 51 in each group was determined to provide 80% power to detect a difference in means of 0.130 (the difference between a Group 1 mean, m1, of 1.020 and a Group 2 mean, m2, of 0.890) assuming that the common standard deviation is 0.230 using a *t*‐test with an alpha‐error of 0.050 and two‐sided significance level. With regard to the power calculation and to compensate for potential dropouts, a total patient number of 120 (60 per group) was defined. In addition, to compensate for early dropouts during the surgical phase, additional patients were incrementally recruited until the target number of successfully placed implants was achieved. Enrolled patients with a single gap received either a two‐piece zirconia implant (test group) (*n* = 60) (*n* = 20, per participating center) or a corresponding conventional two‐piece titanium implant (control group) (*n* = 60) (*n* = 20 per participating center). The random allocation sequence was generated by a professional statistician using computer‐based block randomization (block size 4), stratified by study center to ensure a 1:1 allocation. A central software system from the Medical University of Graz (Randomizer for clinical trials) implemented the sequence and released assignments only after patient enrollment, maintaining allocation concealment.

Data sets from all three centers were pooled, and statistics were performed by a specialized statistician from the Medical University of Graz, Austria. All analyses were performed using SPSS software (version 29), applying a significance threshold of 0.05. Descriptive and exploratory statistical analyses were conducted to examine differences between the two groups. The Kolmogorov–Smirnov goodness‐of‐fit test was employed to assess the normality of data distributions. Intergroup comparisons of continuous outcomes were performed using Linear Mixed‐Effects Models, with the group, gender, and smoking status as fixed effects, center as a random effect, and age as a covariate to account for potential confounding. Categorical variables were analyzed using the chi‐square or Fisher's exact test. Survival rates were compared using Kaplan–Meier curves and the log‐rank test. Details on the specific significance tests applied to each data set are provided in the corresponding tables and figures. Comparisons among the three centers were performed using the chi‐square test, one‐way ANOVA, or Kruskal–Wallis test, depending on the distribution of the respective variables.

## 3 Results

### 3.1 Study Subject Description

Initially, 129 patients, including equal numbers of males (50.4%) and females (49.6%) with a mean age of 53.3 years, were enrolled at 3 different study centers in Basel, Graz, and Zürich (Table 1). Provisional restorations were performed only for 7.8% of the study population. CBCT was performed for 65.9% of the subjects. A detailed description of missing teeth and the number of subjects was prepared. Most subjects lacked teeth at positions 36 and 46, followed by positions 35, 15, and 26, according to the Fédération Dentaire Internationale/International Organization for Standardization (FDI/ISO) tooth numbering system (Table 1). The majority of the patients neither smoked (62%) nor showed any specific complications (99.2%) (Table 1), thereby presenting lower risks associated with the procedure. *p*‐values for comparisons of demographics and baseline characteristics between centers were calculated to assess potential center effects (Table 1, details in Table S1).

**TABLE 1:** Demographics and baseline characteristics of the subjects.

Implant	Basel (*n* = 47)	Graz (*n* = 41)	Zürich (*n* = 41)	Total (*n* = 129)
Titanium, *n* (%)	24 (51.1)	21 (51.2)	21 (51.2)	66 (51.2)
Zirconia, *n* (%)	23 (48.9)	20 (48.8)	20 (48.8)	63 (48.8)

Variable	Titanium	Zirconia	Total	*p* center
Age at implantation (years), mean ± SD (range)	54.0 ± 13.2 (25–82)	52.6 ± 13.1 (25–27)	53.3 ± 13.2 (25–82)	0.002
Gender, *n* (%)				
Male	37 (43.9)	28 (44.4)	65 (50.4)	0.797
Female	29 (56.1)	35 (55.6)	64 (49.6)	
CBCT, *n* (%)				
Yes	42 (63.6)	43 (68.2)	85 (65.9)	< 0.001
No	24 (36.4)	20 (31.7)	44 (34.1)	
Missing tooth, *n* (%)				
11	1 (1.5)		1 (0.8)	
12		1 (1.6)	1 (0.8)	
14	4 (6.1)	3 (4.8)	7 (5.4)	
15	7 (10.6)	4 (6.3)	11 (8.5)	
16	4 (6.1)	3 (4.8)	7 (5.4)	
17		1 (1.6)	1 (0.8)	
23		1 (1.6)	1 (0.8)	
24	5 (7.6)	1 (1.6)	6 (4.7)	
25	2 (3)	5 (7.9)	7 (5.4)	
26	5 (7.6)	5 (7.9)	10 (7.8)	
27	1 (1.5)		1 (0.8)	
35	6 (9.1)	6 (9.5)	12 (9.3)	
36	17 (25.8)	15 (23.8)	32 (24.8)	
37	1 (1.5)		1 (0.8)	
44	1 (1.5)	1 (1.6)	2 (1.6)	
45	1 (1.5)	2 (3.2)	3 (2.3)	
46	10 (15.2)	15 (23.8)	25 (19.4)	
47	1 (1.5)	1 (1.6)	1 (0.8)	
Provisional restoration *n* (%)				
Yes	7 (10.8)	3 (4.8)	10 (7.8)	0.520
No	58 (89.2)	60 (95.2)	118 (92.2)	
Risk factors, *n* (%)				
Smoking				
Former smoker	14 (21.2)	19 (30.2)	33 (25.6)	0.002
Non‐smoker	45 (68.2)	35 (55.6)	80 (62.0)	
Smoker	7 (10.6)	9 (14.3)	16 (12.4)	
Cigarettes per day as *n* (mean)	5 (5.2)	9 (5.6)	14 (5.5)	0.343
Number of years as smoker as *n* (mean)		6 (20.0)	6 (20.0)	0.999
Pack per years score as *n* (mean)	4 (0.2)	8 (5.0)	12 (3.4)	0.065
Packyears as *n* (mean)	3 (11.4)	6 (11.1)	9 (11.3)	0.999
Other complications, *n* (%)				
No	66 (100)	62 (98.4)	128 (99.2)	0.594
Yes		1 (1.6)	1 (0.8)	

Out of 129 patients, seven were excluded for different reasons, for example, implantation not possible in certain patients, patients failed to report a condition under exclusion criteria at initial enrollment, dropout or withdrawal of consent, or placement of an implant other than a test or control implant. Therefore, 122 patients were implanted with 61 patients each for titanium and zirconia implants (Table 2, Figure 2). At the time of suture removal (visit 4), one patient from the zirconia group dropped out due to COVID‐19, resulting in 121 patients. At visit 5a, during the final impression, another two patients were withdrawn due to pregnancy, thus resulting in 119 patients. Two implants were lost before the permanent crown insertion (visit 5b), resulting in 117 patients being prosthetically restored. One patient did not participate in the baseline visit (visit 5c) but was present for the 1‐year follow‐up at visit 6, therefore reducing the number of patients to 116 for visit 5c. During the final 1‐year follow‐up after crown insertion, another 4 patients were lost to follow‐up, and one due to death. Therefore, the final data was obtained from 112 patients (Figure 2), where 57 were implanted with zirconia and 55 with titanium implants. The resulting dropout rate was within the expected range and did not compromise the statistical power or validity of the findings. Baseline characteristics of the 17 subjects who discontinued the study (mean age 55.3 ± 17.2 years; 10 male, 7 female) have been added to Table S2.

**TABLE 2:** Parameters measured on the day of implantation (day 0, visit 2).

	Group	Yes	No	*p* ^a^
		*n* (%)	*n* (%)	
Implant placed	Titanium	61 (100)		
	Zirconia	61 (100)		
Periosteal releasing	Titanium	26 (42.6)	35 (57.4)	0.573
	Zirconia	26 (42.6)	35 (57.4)	
Single tooth X‐ray	Titanium	61 (100)		
	Zirconia	61 (100)		
GBR	Titanium	7 (11.5)	54 (88.5)	0.500
	Zirconia	8 (13.1)	53 (86.9)	
Primary stability	Titanium	60 (98.4)	1 (1.6)	0.500
	Zirconia	61 (100)	0 (0)	
Healing submerged	Titanium	25 (41)	36 (59)	0.288
	Zirconia	21 (34.4)	40 (65.6)	
Any complications	Titanium	1 (1.6)	60 (98.4)	0.016
	Zirconia	8 (13.1)	53 (86.9)	

Abbreviation: GBR, Guided Bone Regeneration.

^a^ Fisher's exact test.

### 3.2 Study Protocol and Interim Measurements

On the day of the implantation (day 0, visit 2), less than half of the patients (42.6%) in each subgroup receiving titanium and zirconia implants underwent periosteal releasing incisions. Similarly, less than 15% of the patients in both the control and test implant groups required GBR. Primary stability after implantation was achieved in all patients except one in the titanium implant group. A similar submerged healing was observed in 41% of the patients with titanium and 34.4% of the patients with zirconia implants. The number of patients with complications during operation was significantly higher in zirconia compared to titanium implant recipients (Table 2). In most of these cases, the connection between the transfer piece and the zirconia implant was lost, and a new implant had to be placed. Importantly, despite these initial complications during implantation, the long‐term survival of the zirconia implants was not compromised.

On the day of suture removal (day 7–14, visit 4), the early wound healing index was measured for most patients. Importantly, most patients in both groups, titanium (100%) and zirconia (96.7%), displayed no complications. Subsequently, after 3–4 months (visit 5a), digital impressions were taken for 61.7% of the titanium and 52.5% of the zirconia implant recipients. Conventional impressions were taken for 88.3% and 100% of the recipients of titanium and zirconia implants, respectively. The crown insertion was done at 4–5 months (visit 5b) and was followed by implant loading at 6 months (visit 5c) and follow‐up after 1 year of crown insertion (visit 6). The marginal bone change as the primary outcome was recorded at implantation, crown insertion, and 1‐year follow‐up. Secondary parameters like plaque index and mesial and distal Papilla Bleeding index (PBI) were recorded at baseline, 3 weeks after implant loading, and 1‐year follow‐up.

### 3.3 Primary Outcome

As shown in Table 3, the mean shoulder‐bone distance was slightly smaller for titanium implants compared to zirconia implants immediately after implantation. Consequently, the titanium implants were placed slightly deeper, but the difference was not significant. The mean ± standard deviation (SD) for MBL from implantation to crown insertion was 1.10 ± 0.78 and 0.94 ± 0.67 mm for titanium and zirconia implants, respectively. The difference between the two groups was not statistically significant. At the time of loading, the measured mean distance between the shoulder and the first bone‐to‐implant contact was 1.76 ± 0.63 mm for titanium implants and 1.79 ± 0.55 mm for zirconia implants (Table 3), indicating that the bone level was at the height of the transition line between the rough and smooth surfaces. There was no further bone loss from the time of crown insertion till 1 year, with a difference of 0.07 ± 0.55 mm and 0.08 ± 0.51 mm for titanium and zirconia implants, respectively (Table 3). Importantly, no significant difference was observed between the control and test implants. The center effect analysis for marginal bone levels and their changes over time is presented in Table S3. When comparing the study centers, it was observed that both titanium (*p* = 0.015) and zirconia implants (*p* = 0.030) were placed significantly deeper in Zurich, where higher bone losses were also recorded compared with the other centers. With regard to bone level changes, significant differences among all centers were detected for titanium implants between implant placement and crown insertion (*p* = 0.002), as well as between Basel and Zurich from implant placement to the 1‐year follow‐up (*p* = 0.021). As differences between centers were observed, a linear mixed‐effects model was applied to compare groups while controlling for center, age, gender and smoking status.

**TABLE 3:** Distances from implant shoulder to first bone‐to‐implant contact and comparison of bone change in mm (MBL) at different time points for titanium and zirconia implants.

	Group	*n*	Mean ± SD	Range	*p* ^a^
Implantation, mesial	Titan	60	0.34 ± 1.18	−2.80 to 3.31	0.335
	ZrO_2_	59	0.69 ± 0.81	−2.10 to 3.05	
Implantation, distal	Titan	60	0.94 ± 0.87	−1.19 to 3.37	0.408
	ZrO_2_	59	1.01 ± 0.70	−0.68 to 2.57	
Implantation, mean	Titan	60	0.64 ± 0.89	−1.68 to 2.90	0.301
	ZrO_2_	59	0.85 ± 0.66	−0.84 to 2.60	
Crown insertion, mesial	Titan	57	1.68 ± 0.80	−1.22 to 3.70	0.743
	ZrO_2_	58	1.68 ± 0.60	−0.30 to 3.45	
Crown insertion, distal	Titan	57	1.83 ± 0.59	0.59 to 3.37	0.385
	ZrO_2_	58	1.90 ± 0.64	0.71 to 3.41	
Crown insertion, mean	Titan	57	1.76 ± 0.63	−0.32 to 3.53	0.799
	ZrO_2_	58	1.79 ± 0.55	0.26 to 3.43	
MBL: Crown insertion, mean − implantation, mean	Titan	57	1.10 ± 0.78	−0.32 to 3.18	0.456
	ZrO_2_	58	0.94 ± 0.67	−0.31 to 3.13	
1 year, mesial	Titan	54	1.71 ± 0.63	−0.55 to 2.96	0.264
	ZrO_2_	57	1.82 ± 0.70	0.73 to 4.13	
1 year, distal	Titan	54	1.93 ± 0.58	0.49 to 3.34	0.818
	ZrO_2_	57	1.95 ± 0.67	0.69 to 3.74	
1 year, mean	Titan	54	1.82 ± 0.55	−0.02 to 3.07	0.474
	ZrO_2_	57	1.89 ± 0.62	0.74 to 3.83	
MBL: 1 year, mean − implantation, mean	Titan	54	1.17 ± 0.97	−0.31 to 4.02	0.444
	ZrO_2_	57	1.02 ± 0.87	−0.50 to 4.38	
MBL: 1 year, mean − crown insertion, mean	Titan	54	0.07 ± 0.55	−0.94 to 2.29	0.818
	ZrO_2_	57	0.08 ± 0.51	−0.74 to 1.50	

*Note:* The distance from the shoulder to the rough–smooth transition line was 1.8 mm for both the test and control implants.

^a^ Linear mixed‐effect model.

### 3.4 Secondary Outcomes

As stated above, along with bone change as the primary outcome, several other parameters like EWHI, probing depth, plaque index, mesial and distal PBI, and implant survival were also measured as secondary outcomes. During the course of the study, two titanium implants were lost, both before prosthetic restoration. One implant failed to achieve osseointegration and was removed following the impression appointment. Another implant exhibited a large circular defect with granulation tissue at the impression appointment and was only osseointegrated in the apical 2 mm; consequently, it was explanted. Both titanium implant losses occurred in the Zurich center, although no statistically significant difference among centers was observed (*p* = 0.128). After accounting for the patients excluded or dropped out during the study, two out of 57 titanium implants were lost, resulting in an implant survival of 96.5% for the titanium group. In contrast, 100% of the zirconia implants survived from implantation till 1 year after crown insertion. The Log‐Rank test yielded a *p*‐value of 0.155, indicating no statistically significant difference between the two survival curves.

Early wound healing index was measured from 120 patients at the time of suture removal (day 7–14, visit 4), where over 85% of the patients in both titanium and zirconia implant groups showed complete flap closure in the range of EHI 1–2 (Table 4). Only a small population exhibited incomplete flap closure with EHI 3–4, with no significant differences among the test (zirconia) and control (titanium) populations. The mean probing depth measured at 1‐year follow‐up after implant loading was 2.78 ± 0.92 mm (range of 1.17 to 5.17 mm) for titanium and 2.89 ± 0.72 mm (range 1.33 to 4.33 mm) for zirconia implants, respectively, with no significant differences between both implant groups (Table 6). However, significant differences between centers were observed (Table S4).

**TABLE 4:** Early wound healing index and occurrence of other complications reported on the day of suture removal (days 7–14, visit 4).

EWHI		*n* (%)	*p* ^a^
EH1 (complete flap closure—no fibrin line in the interproximal area)	Titanium	30 (49.2)	0.570
	Zirconia	33 (55.9)	
EH2 (complete flap closure—fine fibrin line in the interproximal area)	Titanium	23 (37.7)	
	Zirconia	18 (30.5)	
EH3 (complete flap closure—fibrin clot in the interproximal area)	Titanium	8 (13.1)	
	Zirconia	7 (11.9)	
EH4 (incomplete flap closure—partial necrosis of the interproximal tissue)	Titanium	0 (0)	
	Zirconia	1 (1.7)	

Abbreviation: EWHI, Early Wound Healing Index.

^a^ Fisher's exact test.

Parameters like plaque index and mesial or distal Papilla Bleeding Index (PBI) were measured at the time of implant loading (Table 5) and 1‐year follow‐up (Table 6). Peri‐implant health was assessed according to the 2017 World Workshop criteria, established by the American Academy of Periodontology and the European Federation of Periodontology health (Berglundh et al. 2018). No plaque was observed in 75.4% and 77.6% of the recipients of titanium and zirconia implants, respectively, at the time of implant loading (Table 5). For only 22% of the patients in both the titanium and zirconia groups, plaque could be recognized by running a probe across the smooth marginal surface of the implant. At the time of implant loading, plaque was visible to the naked eye for only one patient who received a titanium implant (Table 5). At 1 year, no plaque was observed in 61.8% and 71.9% of the recipients of titanium and zirconia implants, respectively (Table 6). Plaque could be recognized by running a probe across the smooth marginal surface of the implant in 38.2% and 24.6% of the patients in the titanium and zirconia groups, respectively. A plaque was visible to the naked eye for only two patients (3.5%) who received zirconia implants (Table 6). Although no significant differences were found between groups at any evaluated time point, significant center effects were observed (Table S4).

**TABLE 5:** Parameters like Plaque index, PBI mesial, and distal measured at functional loading of the implant (visit 5c).

	Titanium	Zirconia	*p* ^a^
	*n* (%)	*n* (%)	
Plaque index			
No plaque (0)	43 (75.4)	45 (77.6)	0.595
Plaque only recognized by running a probe across the smooth marginal surface of the implant (1)	13 (22.8)	13 (22.4)	
Plaque can be seen by the naked eye (2)	1 (1.8)	0 (0)	
PBI mesial			
No bleeding (0)	39 (68.4)	45 (77.6)	0.595
A single discrete bleeding point (1)	12 (21.1)	10 (17.2)	
Several isolated bleeding points or a single line of blood appears (2)	5 (8.8)	3 (5.2)	
The interdental triangle fills with blood shortly after probing (3)	1 (1.8)	0 (0)	
PBI distal			
No bleeding (0)	42 (75.0)	47 (82.5)	0.595
A single discrete bleeding point (1)	13 (23.2)	9 (15.8)	
Several isolated bleeding points or a single line of blood appears (2)	1 (1.8)	1 (1.8)	

Abbreviation: PBI, Papilla Bleeding Index.

^a^ Chi‐square test.

**TABLE 6:** Performance parameters for titanium and zirconia implants measured at 12‐month follow‐up from the time of implant loading (visit 6).

	Titanium	Zirconia	*p* ^a^
Probing (mean in mm)			0.532
*n*	54	56	
Mean ± SD	2.78 ± 0.92	2.89 ± 0.72	
Range	1.17 to 5.17	1.33 to 4.33	

	*n* (%)	*n* (%)	*p* ^b^
Plaque index			
No plaque (0)	34 (61.8)	41 (71.9)	0.134
Plaque only recognized by running a probe across the smooth marginal surface of the implant (1)	21 (38.2)	14 (24.6)	
Plaque can be seen by the naked eye (2)	0 (0)	2 (3.5)	
PBI mesial			
No plaque (0)	34 (61.8)	29 (50.9)	0.080
A single discrete bleeding point (1)	13 (23.6)	25 (43.9)	
Several isolated bleeding points or a single line of blood appears (2)	7 (12.7)	3 (5.3)	
The interdental triangle fills with blood shortly after probing (3)	1 (1.8)	0 (0)	
PBI distal			
No plaque (0)	33 (60.0)	26 (48.1)	0.224
A single discrete bleeding point (1)	13 (23.6)	20 (37.0)	
Several isolated bleeding points or a single line of blood appears (2)	7 (12.7)	8 (14.8)	
The interdental triangle fills with blood shortly after probing (3)	2 (3.6)	0 (0)	

^a^ Linear mixed‐effect model.

^b^ Chi‐square test.

At implant loading, more than 85% of recipients of both titanium and zirconia implants showed no bleeding or a single discreet bleeding point on the mesial side (Table 5). At 1‐year follow‐up, 61.8% and 50.9% of recipients of titanium and zirconia implants, respectively, showed no mesial bleeding (PBI 0). Another 23.6% and 43.9% of recipients of titanium and zirconia implants, respectively, showed a single discreet bleeding point on the mesial side (PBI 1). Several isolated bleeding points or a single line of blood (PBI 2) were observed in 12.7% and 5.3% of the patients with titanium and zirconia implants, respectively. The interdental triangle filled with blood shortly after probing (PBI 3) in the case of only one patient in the titanium group (Table 6). Similarly, on the distal side, only 1.8% of the patients showed several isolated bleeding points or a single line of blood (PBI 2) at implant loading at 6 months (Table 5). Further, at 1‐year follow‐up, a distal PBI of 0–1 was observed for 83.6% and 85.1% of the patients with titanium and zirconia implants, respectively. Other 12.7% and 14.8% of patients with titanium and zirconia implants, respectively, showed a papilla bleeding index of 2. A PBI 3 was observed in 2 patients in the titanium group (Table 6). No significant differences were observed among the test and control implant groups for PBI at both measured time points and locations, but significant center effects were detected for titanium (Table S2).

## 4 Discussion

This study aimed to evaluate the clinical outcomes of two‐piece zirconia implants with screw‐retained abutments in comparison to the gold standard of titanium implants. The results of this study demonstrated that no statistically significant differences were observed between the two‐piece zirconia implants with a screw‐retained abutment connection and the well‐established titanium implants: (i) No statistically significant differences in marginal bone level changes over the 1‐year postoperative period were observed between the two groups; (ii) zirconia implants exhibited a 100% survival rate, while titanium implants showed a survival rate of 96.5%; (iii) no significant differences were observed between the groups regarding early wound healing, plaque index, papilla bleeding index, or implant survival.

Our findings demonstrate that both titanium and zirconia implants maintain stable marginal bone levels over time, with minimal differences in bone loss. The mean MBL between implantation and crown insertion was 1.10 ± 0.78 mm for titanium implants and 0.94 ± 0.67 mm for zirconia implants, which aligns with previous studies reporting no statistically significant differences in bone remodeling between these materials when implants were placed subcrestally (Rohr et al. 2021). After initial bone remodeling, the marginal bone level in both groups stabilized at the transition line between the rough and smooth implant surface. This was also reflected in the comparison among study centers, where greater bone remodeling was observed in the center in which implants were placed at the deepest level. The negligible bone loss observed between loading and 1 year—0.07 ± 0.55 mm for titanium and 0.08 ± 0.51 mm for zirconia further supports their long‐term viability. This finding is particularly relevant given that titanium implants have been extensively studied and are considered the gold standard in implant dentistry, while no randomized clinical trial data has previously been available for this type of two‐piece zirconia implant. The bone stability and the 100% survival rate observed in this study further support zirconia as a promising alternative for patients seeking a metal‐free implant solution.

Comparisons with other studies on zirconia implants reinforce these observations. One uncontrolled study assessed the same two‐piece zirconia implant system and found a 100% survival rate with low plaque accumulation and shallow probing depths (2.49 ± 0.49 mm) at 15 months. However, no radiographic assessment of the marginal bone level was performed (Lorenz et al. 2022). Similarly, another uncontrolled study using the same implant material and surface characteristics, but in a monotype design, reported outcomes over a 5‐year observation period, with a high survival rate of 97.7% and mean bone loss limited to 0.99 ± 0.59 mm (Gahlert et al. 2022). A systematic review and meta‐analysis study reported a 5‐year zirconia implant survival rate of 97.2%, with a mean MBL at 1.1 mm, reinforcing zirconia's comparable performance to titanium implants (Roehling et al. 2023). In a direct 1‐year comparison between zirconia and titanium implants, a recent study found no statistically significant differences in marginal bone level changes, with 0.1 ± 0.4 mm for zirconia and −0.1 ± 0.7 mm for titanium, demonstrating a non‐significantly different remodeling process. Additionally, patients in this study expressed a preference for zirconia due to its superior soft tissue color, highlighting an esthetic advantage that may be significant in clinical decision‐making (Zuercher et al. 2024). Similarly, Mohseni et al. found that patient‐reported outcomes exceeded 90% satisfaction for both zirconia and titanium implants. The superior esthetic properties of zirconia, particularly in the anterior region, make it a favorable option for patients seeking a more natural appearance (Mohseni et al. 2024).

In some studies, zirconia is considered to offer soft tissue benefits. For instance, one preclinical study examined cell adhesion and proliferation on structured abutment materials and found that fibroblasts proliferated more effectively on zirconia than on titanium, thereby allowing better healing (Nothdurft et al. 2015). Furthermore, in an experimental study, Thoma et al. reported significantly less loss of peri‐implant mucosal height with zirconia compared to titanium implants when buccal bone dehiscence was present (Thoma et al. 2015). In addition, one meta‐analysis reviewed over 30 preclinical and clinical studies on dental implants and abutment materials, focusing on their impact on peri‐implant health and the prevention of peri‐implantitis. The results showed a statistically significant advantage of zirconia implants over titanium in promoting a favorable alveolar bone response (Haugen and Chen 2022). In a randomized clinical study, one titanium implant and one zirconia implant were placed adjacent to each other and left without cleaning for 3 weeks. Under these experimentally induced mucositis conditions, the zirconia implants exhibited lower plaque accumulation and bleeding scores compared to the titanium implants (Bienz et al. 2021). In contrast to these promising studies and analyses, our study found no statistically significant differences in plaque index, bleeding on probing, probing depth, and not even in early wound healing index between zirconia and titanium implants, indicating no clinical superiority in peri‐implant tissue responses. This is in line with another study with a small sample size of 16 zirconia and 15 titanium implants, but with a long follow‐up of over 80 months. They demonstrated that two‐piece zirconia implants exhibit comparable long‐term performance to titanium implants. Despite the loss of two zirconia and one titanium implant, the remaining implants were stable, with no fractures or prosthetic complications. Like our study, this study also reported no significant differences in plaque index, bleeding on probing, or marginal bone loss (Koller et al. 2020). Although no clinically significant superiority in the peri‐implant soft tissue biological response could be demonstrated, the present study supports the viability of zirconia as an alternative to titanium, even over extended observation periods. This is further reinforced by a recent systematic review, which confirmed the long‐term reliability of zirconia implants, reporting a 10‐year cumulative survival rate of 95.1% and low early marginal bone loss values, underscoring their clinical success over time (Mohseni et al. 2024).

A limitation of the investigated zirconia implant in this study was the higher incidence of intraoperative complications, primarily due to disconnection between the implant and the transfer piece. This issue may be related to the design of the pick‐up connection and highlights the need for greater surgical caution compared to titanium implants. As this technical aspect has not yet been reported in the literature, it represents a clinically relevant observation for practitioners using this implant system. However, despite the increased intraoperative handling requirements, the 1‐year clinical performance of the zirconia implants was not compromised.

In summary, the present study confirms that both titanium and zirconia implants demonstrate high survival rates, low biological complications, and stable marginal bone levels over 1 year. When compared with existing literature, the findings reinforce the clinical reliability of both materials, with zirconia implants offering potential advantages in soft tissue response and esthetics. The findings of this study also indicate that the modified implant shoulder design and the use of a titanium screw within the zirconia core do not compromise implant stability or biological integration while offering increased prosthetic flexibility. Moreover, this study contributes to the limited scientific evidence available on two‐piece screw‐retained zirconia implants in humans. While the large patient sample represents a strength of this study, the relatively short follow‐up period must be considered a major limitation. In addition, blinding of the outcome assessor was not feasible due to the clearly distinguishable clinical and radiographic appearance of the two implant materials, which may introduce a risk of information bias. Moreover, data were collected in university settings in Switzerland and Austria, which may limit the external validity of the findings.

Given the increasing demand for metal‐free dental implants from patients with metal sensitivities, this study provides strong evidence supporting the safety and functional success of two‐piece zirconia implants, paving the way for their broader clinical use. Future studies should focus on optimizing zirconia implant designs and surface modifications to enhance mechanical durability and long‐term success. Further randomized controlled trials with extended follow‐up periods will be necessary to refine implant selection criteria and optimize patient outcomes.

## 5 Conclusion

This study demonstrated that no statistically significant differences were observed between the two‐piece zirconia implant with a screw‐retained abutment connection and the well‐established two‐piece titanium implant in terms of marginal bone stability, implant survival, and peri‐implant health over a 1‐year follow‐up. The absence of significant differences in plaque index, papilla bleeding index, and bone‐level changes between the two materials supports the viability of zirconia as an alternative for dental implants.

Additionally, despite initial technical challenges, the 100% survival rate of zirconia implants suggests that the modified implant design does not compromise mechanical stability or biological integration. Supporting evidence from human and animal studies further confirms that two‐piece zirconia implants achieve osseointegration and clinical success rates without statistically significant differences to titanium implants.

Given the increasing demand for metal‐free dental solutions, these findings provide strong clinical evidence supporting the long‐term stability and functional success of two‐piece zirconia implants. However, long‐term studies with larger sample sizes and extended follow‐up periods are warranted to validate these findings and optimize clinical protocols for zirconia implant applications.

## Author Contributions

S.K., M.P., and R.E.J. conceived the idea, and M.B. and S.K. led the writing. S.K., M.P., M.B., and R.E.J. performed the surgeries. S.K., M.P., M.B., A.P., and R.E.J. performed the prosthetics. S.K., M.P., V.H., and M.B. collected, analyzed, and interpreted the data. All authors critically reviewed and approved the final version of the manuscript.

## Funding

The implants and other materials, such as membranes, healing abutments, impression abutments, and abutments for the final crown, were provided by the Institut Straumann AG (Basel, Switzerland). The bone substitute material was sponsored by Geistlich Pharma AG (Wollhusen, Switzerland). This investigator‐initiated trial was funded by the International Team for Implantology association (Grant No: 1218_2017). All scientific decisions—including study design, conduct, analysis, and publication—were made independently by the investigators.

## Conflicts of Interest

M.B. reports grants from International Team for Implantology (ITI), Straumann, VITA, Prosec, Geistlich, and Osteology during the conduct of the study. V.H. reports grants from ITI, grants from Straumann, outside the submitted work. R.E.J. reports grants from ITI and Osteology, and grants, personal fees, and others from Geistlich, Straumann, VITA, Henry Schein, and TRI during the conduct of the study. The other authors report no conflicts of interest.

## Supporting information


**Data S1:** clr70094‐sup‐0001‐supinfo01.docx.

## Data Availability

The data that support the findings of this study are available on request from the corresponding author. The data are not publicly available due to privacy or ethical restrictions.
