# Supplementary material for: One‐Year Analysis of Clinical and Radiological Outcomes of Two‐Piece Zirconia Compared to Titanium Implants: A Multicenter Prospective Randomized Clinical Trial
Source: Clin Oral Implants Res. 2026 Jan 22;37(4):496–508. doi: 10.1111/clr.70094 (PMC13051413; doi:10.1111/clr.70094)
Supplement: Supplementary file 1 — Data S1: clr70094‐sup‐0001‐supinfo01.docx. [file CLR-37-496-s001.docx]

**Supplementary Table 1. Analysis of center effects on demographics and baseline characteristics**

age * center

|  | min | max | mean | SD | p-value* |
| --- | --- | --- | --- | --- | --- |
| Basel | 25 | 82 | 56,4 | 13,2 | p = 0,002 |
| Graz | 26 | 72 | 47,4 | 12,9 |  |
| Zürich | 27 | 74 | 55,7 | 11,5 |  |

**One-way Anova*

gender * center

|  | female | % | male | % | p-value* |
| --- | --- | --- | --- | --- | --- |
| Basel | 23 | 49 | 24 | 51 | p = 0,787 |
| Graz | 22 | 54 | 19 | 46 |  |
| Zürich | 19 | 46 | 22 | 54 |  |

**Chi Square test*

CBCT * center

|  | yes | % | no | % | p-value* |
| --- | --- | --- | --- | --- | --- |
| Basel | 47 | 100 | 0 | 0 | p < 0,001 |
| Graz | 20 | 49 | 21 | 51 |  |
| Zürich | 18 | 44 | 23 | 56 |  |

**Chi Square test*

Provisional Restoration * center

|  | yes | % | no | % | p-value* |
| --- | --- | --- | --- | --- | --- |
| Basel | 2 | 4,3 | 45 | 95,7 | p = 0,520 |
| Graz | 4 | 10 | 36 | 90 |  |
| Zürich | 4 | 10 | 37 | 90 |  |

**Chi Square test*

Other complications * center

|  | yes | % | no | % | p-value* |
| --- | --- | --- | --- | --- | --- |
| Basel | 1 | 2,5 | 39 | 97,5 | p = 0,594 |
| Graz | 1 | 2,5 | 39 | 97,5 |  |
| Zürich | 0 | 0 | 41 | 0 |  |

**Chi Square test*

Smoking * center

|  | former smoker | | never smoked | | Smoker | |  |
| --- | --- | --- | --- | --- | --- | --- | --- |
|  | n | % | n | % | n | % | p-value* |
| Basel | 14 | 30 | 29 | 62 | 4 | 8 | p = 0,002 |
| Graz | 7 | 17 | 33 | 81 | 1 | 2 |  |
| Zürich | 12 | 29 | 18 | 44 | 11 | 27 |  |

**Chi Square test*

**Supplementary Table 2.  Baseline characteristics of the 17 subjects who discontinued the study.**

| Study interval | n | Center (Basel / Graz / Zürich) | Material (ZrO₂ / Titanium) | Age (years) | Male | Female | Implant position(s) |
| --- | --- | --- | --- | --- | --- | --- | --- |
| Visit 1 → Visit 2 | 7 | 6 / 1 / – | 2 / 5 | 54, 60, 62, 62, 65, 73, 77 | 4 | 3 | 12, 23, 24, 25, 25, 26, 46 |
| Visit 2 → Visit 4 | 1 | 1 / – / – | 1 / 0 | 30 | 0 | 1 | 36 |
| Visit 4 → Visit 5a | 2 | – / 2 /– | 1 / 1 | 31, 32 | 0 | 2 | 16, 36 |
| Visit 5a → Visit 5b | 2 | – / – / 2 | 1 / 1 | 62, 69 | 2 | 0 | 25, 26 |
| Visit 5b → Visit 5c | 1 | – / – / 1 | 0 / 1 | 50 | 1 | 0 | 46 |
| Visit 5c → Visit 6 | 4 | 2 / – / 2 | 1 / 1 | 66, 80, 31, 36 | 3 | 1 | 15, 35, 36, 45 |
| Total | 17 | 9 / 3 / 5 | 6 / 9 | Mean 55.3 (± 17.2) | 10 | 7 |  |

**Supplementary Table 3. Center effect analysis for marginal bone level (MBL, mm) and comparison of changes over time for titanium and zirconia implants.**

|  | **material** | **center** | **n** | **min** | **max** | **mean** | **SD** | **p-value*** |
| --- | --- | --- | --- | --- | --- | --- | --- | --- |
| **Implantation** | Titanium | Basel | 19 | -1,68 | 2,16 | 1,11 | 0,87 | p = 0,015 |
|  |  | Graz | 20 | -1,41 | 1,48 | 0,51 | 0,67 |  |
|  |  | Zürich | 21 | -1,08 | 2,90 | 0,34 | 0,95 |  |
|  | ZrO2 | Basel | 19 | 0,12 | 1,79 | 1,12 | 0,43 | p = 0,030 |
|  |  | Graz | 20 | 0,01 | 1,71 | 0,86 | 0,39 |  |
|  |  | Zürich | 20 | -0,84 | 2,60 | 0,57 | 0,92 |  |
| **Crown Insertion** | Titanium | Basel | 19 | -0,32 | 2,43 | 1,72 | 0,57 | p = 0,888 |
|  |  | Graz | 19 | 0,67 | 2,80 | 1,74 | 0,57 |  |
|  |  | Zürich | 19 | 0,21 | 3,53 | 1,82 | 0,76 |  |
|  | ZrO2 | Basel | 19 | 0,90 | 3,43 | 1,80 | 0,57 | p = 0,728 |
|  |  | Graz | 19 | 0,71 | 2,46 | 1,86 | 0,51 |  |
|  |  | Zürich | 20 | 0,26 | 2,59 | 1,72 | 0,59 |  |
| **MBL: Crown Insertion - Implantation** | Titanium | Basel | 19 | -0,27 | 2,02 | 0,61 | 0,53 | p = 0,002 |
|  |  | Graz | 19 | -0,32 | 2,43 | 1,24 | 0,66 |  |
|  |  | Zürich | 19 | -0,02 | 3,18 | 1,45 | 0,88 |  |
|  | ZrO2 | Basel | 19 | -0,06 | 1,85 | 0,68 | 0,51 | p = 0,080 |
|  |  | Graz | 19 | -0,31 | 2,19 | 1,00 | 0,65 |  |
|  |  | Zürich | 20 | -0,18 | 3,13 | 1,15 | 0,76 |  |
| **1y** | Titanium | Basel | 19 | 1,03 | 2,55 | 1,82 | 0,38 | p = 0,793 |
|  |  | Graz | 18 | 0,58 | 3,07 | 1,76 | 0,55 |  |
|  |  | Zürich | 17 | -0,02 | 2,94 | 1,89 | 0,70 |  |
|  | ZrO2 | Basel | 19 | 1,04 | 3,83 | 1,87 | 0,63 | p = 0,952 |
|  |  | Graz | 19 | 0,74 | 3,11 | 1,87 | 0,55 |  |
|  |  | Zürich | 19 | 0,90 | 3,54 | 1,92 | 0,70 |  |
| **MBL: 1y - Impl** | Titanium | Basel | 19 | -0,31 | 3,65 | 0,71 | 0,94 | p = 0,021 |
|  |  | Graz | 18 | 0,14 | 3,21 | 1,27 | 0,73 |  |
|  |  | Zürich | 17 | -0,16 | 4,02 | 1,58 | 1,07 |  |
|  | ZrO2 | Basel | 19 | -0,37 | 2,25 | 0,74 | 0,68 | p = 0,135 |
|  |  | Graz | 19 | -0,44 | 2,33 | 1,01 | 0,60 |  |
|  |  | Zürich | 19 | -0,50 | 4,38 | 1,30 | 1,16 |  |
| **MBL: 1y - Crown Insertion** | Titanium | Basel | 19 | -0,94 | 2,29 | 0,10 | 0,64 | p = 0,940 |
|  |  | Graz | 18 | -0,78 | 1,02 | 0,03 | 0,47 |  |
|  |  | Zürich | 17 | -0,79 | 0,96 | 0,08 | 0,55 |  |
|  | ZrO2 | Basel | 19 | -0,63 | 1,38 | 0,07 | 0,45 | p = 0,614 |
|  |  | Graz | 19 | -0,67 | 1,33 | 0,01 | 0,51 |  |
|  |  | Zürich | 19 | -0,74 | 1,50 | 0,17 | 0,59 |  |

*One-Way Anova

**Supplementary Table 4. Center effect on probing depth, Papilla Bleeding Index (PBI), and Plaque Index for titanium and zirconia implants at the 1-year follow-up.**

| **Probing** | group | center | n | min | max | mean | SD | p-value* | |
| --- | --- | --- | --- | --- | --- | --- | --- | --- | --- |
|  | Titanium | Basel | 19 | 1,50 | 5,17 | 2,48 | 0,84 | p < 0,001 | |
|  |  | Graz | 19 | 1,17 | 3,67 | 2,44 | 0,74 |  | |
|  |  | Zürich | 16 | 1,67 | 4,67 | 3,55 | 0,75 |  | |
|  | ZrO2 | Basel | 19 | 1,50 | 3,33 | 2,59 | 0,45 | p < 0,001 | |
|  |  | Graz | 19 | 1,33 | 3,83 | 2,60 | 0,77 |  | |
|  |  | Zürich | 18 | 2,33 | 4,33 | 3,52 | 0,50 |  | |
| *One-Way Anova |  |  |  |  |  |  |  |  |  |

|  | **Basel** | | **Graz** | | **Zürich** | |  |
| --- | --- | --- | --- | --- | --- | --- | --- |
| **PBI distal Titanium** | n | % | n | % | n | % | p-value* |
| No bleeding (0) | 18 | 0,90 | 15 | 0,83 | 9 | 0,50 | p = 0,012 |
| A single discreet bleeding point (1) | 1 | 0,05 | 3 | 0,17 | 9 | 0,50 |  |
| Several isolated bleeding points or a single line of blood appears (2) | 1 | 0,05 |  |  |  |  |  |

| **PBI mesial Titanium** | n | % | n | % | n | % | p-value* |
| --- | --- | --- | --- | --- | --- | --- | --- |
| No bleeding (0) | 15 | 78,9 | 11 | 57,9 | 8 | 47,1 | p = 0,088 |
| A single discreet bleeding point (1) | 2 | 10,5 | 3 | 15,8 | 8 | 47,1 |  |
| Several isolated bleeding points or a single line of blood appears (2) | 2 | 10,5 | 4 | 21,1 | 1 | 5,9 |  |
| The interdental triangle fills with blood shortly after pr. (3) |  |  | 1 | 5,3 |  |  |  |

| **PBI distal ZrO2** | n | % | n | % | n | % | p-value* |
| --- | --- | --- | --- | --- | --- | --- | --- |
| No bleeding (0) | 20 | 1,00 | 12 | 0,71 | 15 | 0,75 | p = 0,078 |
| A single discreet bleeding point (1) |  |  | 4 | 0,24 | 5 | 0,25 |  |
| Several isolated bleeding points or a single line of blood appears (2) |  |  | 1 | 0,06 |  |  |  |

| **PBI mesial ZrO2** | n | % | n | % | n | % | p-value* |
| --- | --- | --- | --- | --- | --- | --- | --- |
| No bleeding (0) | 10 | 52,6 | 12 | 63,2 | 7 | 36,8 | p = 0,235 |
| A single discreet bleeding point (1) | 9 | 47,4 | 5 | 26,3 | 11 | 57,9 |  |
| Several isolated bleeding points or a single line of blood appears (2) |  |  | 2 | 10,5 | 1 | 5,3 |  |

| **Plaque index Titanium** | n | % | n | % | n | % | p-value* |
| --- | --- | --- | --- | --- | --- | --- | --- |
| No plaque (0) | 16 | 0,84 | 13 | 0,68 | 5 | 0,29 | p = 0,003 |
| Plaque only recognized by running a probe across the smooth marginal surface of the implant (1) | 3 | 0,16 | 6 | 0,32 | 12 | 0,71 |  |

| **Plaque index ZrO2** | n | % | n | % | n | % | p-value* |
| --- | --- | --- | --- | --- | --- | --- | --- |
| No plaque (0) | 17 | 0,90 | 15 | 0,79 | 9 | 0,47 | p = 0,003 |
| Plaque only recognized by running a probe across the smooth marginal surface of the implant (1) | 2 | 0,11 | 2 | 0,11 | 10 | 0,53 |  |
| Plaque can be seen by the naked eye (2) |  |  | 2 | 0,11 |  |  |  |

** Chi-Square test*
